# Application of Hybridization Chain Reaction/CRISPR-Cas12a for the Detection of SARS-CoV-2 Infection

**DOI:** 10.3390/diagnostics13091644

**Published:** 2023-05-07

**Authors:** Kate Obaayaa Sagoe, Mutinda Cleophas Kyama, Naomi Maina, Moses Kamita, Muturi Njokah, Kelvin Thiong’o, Bernard N. Kanoi, Ernest Apondi Wandera, Davies Ndegwa, Dickson Mwenda Kinyua, Jesse Gitaka

**Affiliations:** 1 Department of Molecular Biology and Biotechnology, Pan African University Institute for Basic Sciences, Technology and Innovation (PAUSTI), Nairobi P.O. Box 62000-00200, Kenya; 2 Department of Medical Laboratory Science, College of Health Sciences, Jomo Kenyatta University of Agriculture & Technology, Nairobi P.O. Box 62000-00200, Kenya; mkyama@jkuat.ac.ke; 3 Department of Biochemistry, College of Health Sciences, Jomo Kenyatta University of Agriculture & Technology, Nairobi P.O. Box 62000-00200, Kenya; 4 Directorate of Research and Innovation, Mount Kenya University, Thika P.O. Box 342-01000, Kenyajgitaka@mku.ac.ke (J.G.); 5 Center for Biotechnology Research and Development, Kenya Medical Research Institute, Nairobi P.O. Box 54840-00200, Kenya; 6 Center for Virus Research, Kenya Medical Research Institute, Nairobi P.O. Box 54840-00200, Kenya; 7 Department of Medical Laboratory Sciences, Kenya Medical Training College, Nairobi P.O. Box 30195-00100, Kenya; 8 Department of Physical Sciences, Meru University of Science & Technology, Meru P.O. Box 972-60200, Kenya; 9 Department of Pure and Applied Sciences, Kirinyaga University, Kerugoya P.O. Box 143-10300, Kenya

**Keywords:** hybridization chain reaction (HCR), severe acute respiratory syndrome coronavirus 2 (SARS-CoV-2), clustered regularly interspaced short palindromic repeats (CRISPR), COVID-19, point-of-care (POC), diagnostics

## Abstract

Globally, the emergence of the coronavirus disease (COVID-19) has had a significant impact on life. The need for ongoing SARS-CoV-2 screening employing inexpensive and quick diagnostic approaches is undeniable, given the ongoing pandemic and variations in vaccine administration in resource-constrained regions. This study presents results as proof of concept to use hybridization chain reaction (HCR) and clustered regularly interspaced short palindromic repeats (CRISPR)/Cas12a complex for detecting SARS-CoV-2. HCR hairpin probes were designed using the NUPACK web-based program and further used to amplify the SARS-CoV-2 N gene in archived nasopharyngeal samples. The results were visualized using agarose gels and CRISPR Cas12a-based lateral flow strips. The assay was evaluated using the gold standard, real-time polymerase chain reaction (RT-PCR), as recommended by the World Health Organization (WHO). The results show the comparative efficiency of HCR to RT-PCR. This study shows that HCR and CRISPR are viable alternatives for diagnosing SARS-CoV-2 in samples.

## 1. Introduction

The World Health Organization (WHO), on 11 March 2020, declared the severe acute respiratory syndrome coronavirus 2 (SARS-CoV-2) as a pandemic [1,2]. The causative virus is a newly discovered pathogenic member of the beta coronavirus genus, which causes the coronavirus disease 2019 (COVID-19). The pathogen has primarily been seen to cause respiratory illnesses, including coughing and shortness of breath. The SARS-CoV-2 virus can bind to the Angiotensin Converting Enzyme 2 (ACE2) receptors found all over the body and cause harm to almost every system, including the lungs, heart, kidney, intestine, and brain [3,4].

As of 12 March 2023, more than 760 million COVID-19 cases had been registered since the first case was identified in December 2019 [3], and more than 6.8 million fatalities had also been recorded globally [5]. The introduction of vaccines towards the end of 2020 has helped reduce the severe illness and mortality witnessed earlier. However, according to data from the COVID-19 global database of vaccinations in March 2023, only 28.5% of residents of under-resourced countries received a minimum of one dose, compared to 69.8% of all people worldwide [6]. With these disparities in the distribution of vaccines in some regions, there is still a high risk of developing highly transmissible SARS-CoV-2 mutants and hence the need for continuous screening and testing.

The emergence of COVID-19 disease has led to the advancement of several molecular and immunological diagnostic techniques for the detection of SARS-CoV-2 [7]. Real-time polymerase chain reaction (RT-PCR) has been the de facto gold-standard diagnostic technique for screening SARS-CoV-2 because of its outstanding precision, specificity, and high sensitivity. However, in resource-limited settings such as Kenya, RT-PCR is still expensive, available only in limited places, and requires qualified personnel [8]. Several point-of-care (POC) diagnostic tools have also been reported, such as recombinase-aided amplification (RAA), loop-mediated isothermal amplification (LAMP), and recombinase polymerase amplification (RPA), which use isothermal amplification techniques, employ simultaneous, reverse transcriptions, and do not need special instruments. However, non-specific amplification is problematic with these approaches [9]. The quest for rapid diagnosis has also led to the creation of commercially available rapid tests, which are mostly COVID-19 antigen-based; however, most have low sensitivity when compared to RT-PCR, limiting their ability to identify asymptomatic persons and obstructing proper viral transmission control [10,11]. 

To address these issues, clustered regularly interspaced short palindromic repeats (CRISPR)-based techniques have been proposed to be a possible diagnostic tool in this area [12,13]. The CRISPR systems can be made of multiple nuclease-active Cas effector proteins that couple with CRISPR RNA (crRNA) or guide RNA (gRNA) and a protospacer flanking sequence (PFS), or protospacer adjacent motif (PAM). Other CRISPR-Cas systems, however, might not require PAM or PFS to choose the sequence target [12,14]. Depending on the sequence and quantity of Cas genes, CRISPR-Cas systems can be divided into two classes and six types. The fundamental difference between the two classes is how their effector modules, which are involved in interference and crRNA processing, are configured. Types I, III, and IV of Class 1 systems demand a multi-subunit effector complex composed of many Cas proteins, but Type II of Class 2 systems only demand a monomeric, multi-domain effector protein (Types II, V, and VI). Type II (Cas 9), Type V (Cas 12), and Type VI (Cas 13) CRISPR-Cas systems are the three most frequently utilized for these diagnostic objectives [15].

CRISPR has shown potential for detecting nucleic acids in point-of-care testing, avoiding the need for costly laboratory materials while allowing speedy and cost-effective detection in RNA and DNA samples of diverse sources [16]. The sequence specificity needed for both the nucleic acid amplification phase and the CRISPR-Cas detection step is what gives CRISPR diagnostic approaches their high sensitivity and specificity [17]. Both lateral flow strips and fluorescence detection can be used in the readout of Cas-mediated nucleic acid probe cleavage, making it suitable for point-of-care diagnostics [18]. With point-of-care tools such as paper-based lateral flow and inexpensive reagents, diagnostic procedures using CRISPR technology can decrease laboratory workload and patient expenditures [14,15]. Several platforms built using CRISPR have been employed in the screening of SARS-CoV-2 infection. Some examples include Specific High-Sensitivity Enzymatic Reporter Unlocking (SHERLOCK), COVID-19 CRISPR-based fluorescent diagnosis system (COVID-19 CRISPR-FDS), as well as SARS-CoV-2 DNA Endonuclease-Targeted CRISPR Trans Reporter (DETECTR) [19,20,21,22]. These platforms, however, employ different enzyme-based steps for amplifying the target nucleic acid before CRISPR-Cas detection, which renders the techniques complex and expensive. 

The use of isothermal non-enzymatic nucleic acid amplification has recently gained attention due to its ability to amplify nucleic acids with high flexibility, comparability readouts, programmability, and low cost [23,24,25,26]. One of many examples of these enzyme-free DNA circuits is the hybridization chain reaction (HCR) that was reported in 2004 [25]. Since then, HCR has been applied to various detection targets where polymerization techniques do not require enzymes and can be performed at room temperature, making it a potentially suitable diagnostic tool [10,27]. An HCR reaction consists mainly of three molecular elements: two fuel hairpins (H1 and H2 probes) and a target gene segment, sometimes called an initiator. The H1 and H2 probes have hairpin-like secondary structures composed of a stem, loop, and an overhang toehold with metastability characteristics [25]. The two metastable DNA hairpins coexist in the HCR buffer—a solution used in HCR reactions—until the first hairpin binds to the initiator. When an initiator is added to the reaction mixture containing the H1 and H2 hairpin probes, the target binds to the H1 hairpin, which opens its sticky fragment that binds to the complementary part in the H2 hairpin. This process causes a cascade of reaction where the opened-up sticky fragment of H2, which is identical to the initial target, opens to the next H1 [28,29]. A nicked double helix is created due to the two DNA hairpins’ sequential assembly process, which sets off a chain reaction that continues to grow until the hairpin supply is depleted. The end result of HCR can be detected using gels or other sensing devices, which eliminates the need for specialist detection equipment [25,29,30]. 

In recent years, combined studies on HCR and CRISPR have been explored for the identification of different targets, including hepatitis and influenza viruses [23,27,30,31,32]. The combination of the two technologies is encouraged by the non-enzymatic nature of HCR and CRISPR’s high specificity and sensitivity. However, there are limited reports on using HCR and CRISPR-Cas12a in diagnosing SARS-CoV-2 infection [33,34]. In this report, HCR and CRISPR-Cas12a were combined to detect SARS-CoV-2 infection. The results from this study could advance our knowledge of the combined HCR/CRISPR-Cas12a as a tool for mass SARS-CoV-2 detection. The combination of HCR and CRISPR-Cas12a could be developed as an alternative diagnostic technology for the detection of other pathogens, showing great promise for point-of-care diagnostics in resource-constrained countries. 

## 2. Materials and Methods

### 2.1. Materials

Following Miti and Zuccheri [28], NUPACK, a web-based application, was used to construct and simulate synthetic target sequences and H1 and H2 hairpin probes to target the SARS-CoV-2 N gene (Table 1). HCR probes and the target were synthesized and purified by Macrogen Incorperated (Amsterdam, The Netherlands). 

The Directorate of Research at Mount Kenya University (Thika, Kenya) provided the archived samples, which were kept in a viral transport medium at −80 °C. The QIAamp Viral RNA Extraction Kit was purchased from Qiagen (Hilden, Germany). Purchases were made from New England Biolabs Incorporated (Ipswich, MA, USA) and Applied Biological Materials Incorporated (Richmond, BC, Canada) for the Protoscript II first-strand cDNA synthesis kit and Safeview Classic dye, respectively. Guide RNA and reporter sequences (Table 1) were purchased from Eurofins (Luxembourg, Luxembourg). The Lateral Flow Strips (Milenia HybriDetect 1) were purchased from TwistDx (Cambridge, UK). Other reagents were purchased from Thermo Fisher Scientific (Waltham, MA, USA) and Inqaba Biotech East Africa Limited (Nairobi, Kenya). 

### 2.2. HCR Probes Testing

The hybridization chain reaction was prepared using H1 and H2 probes and a synthetic N gene segment as the target, as previously described [28,29]. Briefly, the stock solution (100 μM) of the target sequence and H1 and H2 probes were diluted in an HCR buffer (0.5 M NaCl, 50 mM Na_2_HPO_4_, pH 6.8) to have a final concentration of 1 μM, 2 μM, 3 μM, 4 μM, and 5 μM. Ten microliters of the target and probes were then individually heated for 5 min at 95 °C and then allowed to cool for 1 hour at ambient temperature. The target, H1, and H2 were combined in a 1:1:1 ratio to make a total reaction volume of 30 μL. The HCR reaction was incubated at 25 °C for an hour. For visualization, 2% agarose gel was prepared using SafeView Classic (Applied Biological Materials Incorporated, British Columbia, Canada) in a 1x TBE buffer (Glentham Life Sciences, Wiltshire, UK), and 10 μL of the product was loaded on the gel and ran at 150 V for 40 min. Imaging was performed using the Vilber E-box visualizer. The presence of a smear indicated a positive result while the absence of a smear indicated a negative result.

### 2.3. Sensitivity Testing of HCR Reaction

To model the detection of a lower concentration of targets, H1 and H2 probes were kept at a ratio of 1:1 while varying the target concentration. Five micromolar of H1 and H2 probes were separately prepared in an HCR buffer. The target was then serially diluted from 10 μM to 0.0781 μM. Amplification and detection using gel electrophoresis were performed as described above (Section 2.2). 

### 2.4. CRISPR-Cas12a Detection

HCR product using 5 μM of the target and each of the two probes were used in CRISPR detection of SARS CoV-2. Before carrying out the LbCas12a trans-cleavage assay, LbCas12a-gRNA complexes were prepared as previously described [21,35]. This preparation was carried out by pre-incubating 5 μM LbCas12a with 1 μM gRNA and NEBuffer 2.1 in a 20 μL reaction volume for 30 min at 38 °C. After pre-incubation, the ssDNA reporter (/56-FAM/TTATTATT/3Bio) was introduced to the reaction. Then, 2 µL of HCR amplicons was combined with 22 µL of the CRISPR/Cas/Reporter mixture and 78 µL of 1× NEBuffer 2.1, which were all incubated at 38 °C for 20 min. Milenia HybriDetect 1 lateral flow strips were then dipped into the reaction tube. The results were observed after two minutes. Positive results were recognized by the test line on the strip’s upper side, while negative results were recognized by the control line on the strip’s bottom side. 

### 2.5. Clinical Evaluation of HCR Reaction

The nasopharyngeal samples used in this study were first confirmed using RT-PCR. Five positive and five negative samples were used for the reaction. Following the manufacturer’s instructions, RNA was extracted using the QIAamp viral RNA (Qiagen, Hilden, Germany) extraction kit from confirmed RT-PCR positive and negative SARS-CoV-2 samples [36]. The Protoscript II first strand cDNA synthesis kit (New England Biolabs Incorporated, Ipswich, MA, USA) was then used to convert the isolated RNA samples to cDNA according to the manufacturer’s quick protocol. cDNA samples were then subjected to HCR. Next, 6 µL of cDNA were diluted in 4 µL of HCR buffer and then reacted with 5 μM of H1 and H2 probes. The specificity assay was carried out using cDNAs of seasonal flu viruses (H1N1pdm09, H3N2 B/Yamata, and B/Victoria) to check for cross-reactivity.

## 3. Results

### 3.1. HCR Probes Testing

**Figure 1:**
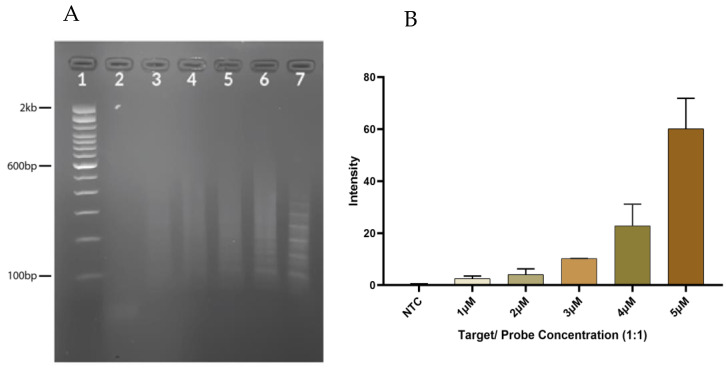
The different concentrations of target and probes. (**A**) Gel image showing the different concentrations of the synthesized target to H1 and H2 probes in a 1:1:1 ratio. Lane 1: 100 bp ladder, Lanes 2–7: NTC, 1 μM, 2 μM, 3 μM, 4 μM, and 5 Μm, respectively. (**B**) Graph of densitometric analysis data expressed as the mean of experiment plotted using GraphPad prism v8 showing variation in band intensities.

Assessment of HCR products showed an increase in the concentration of probes, and the target increased the band intensity (Figure 1). Five-micromolar (5 μM) probes were determined to be the optimum concentration and used for the rest of the experiments.

### 3.2. Sensitivity Testing of HCR Reaction

**Figure 2:**
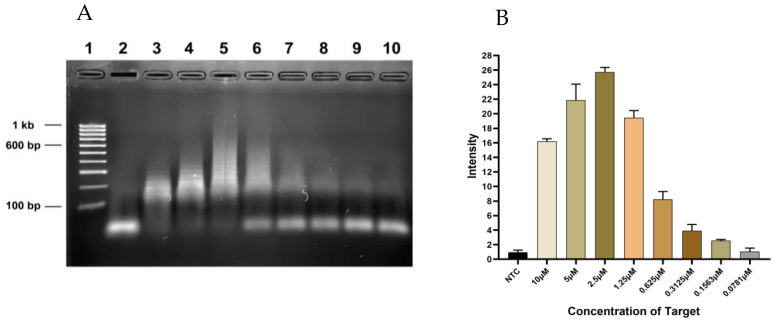
The sensitivity of HCR assay. (**A**) Gel image showing twofold serial dilution of 10 μM of the target to 5 μM of probes. Lane 1: 100 bp ladder, Lanes 2–10: NTC, 10 μM, 5 μM, 2.5 μM, 1.25 μM, 0.625 μM, 0.3125 μM, 0.1563 μM, 0.0781 μM. (**B**) Graph of densitometric analysis data expressed as the mean of experiment plotted using GraphPad showing a pattern of decreasing concentration of target to probe.

In the limit of detection modeling of the target, visualization of the chain reaction reduced with a reduction in the target concentration (Figure 2A). According to the densitometric analysis, the observed limit of detection was 0.1563 µM since the intensity at 0.0781 µM was similar to that of the NTC (Figure 2B). 

### 3.3. CRISPR-Cas12a Detection

**Figure 3:**
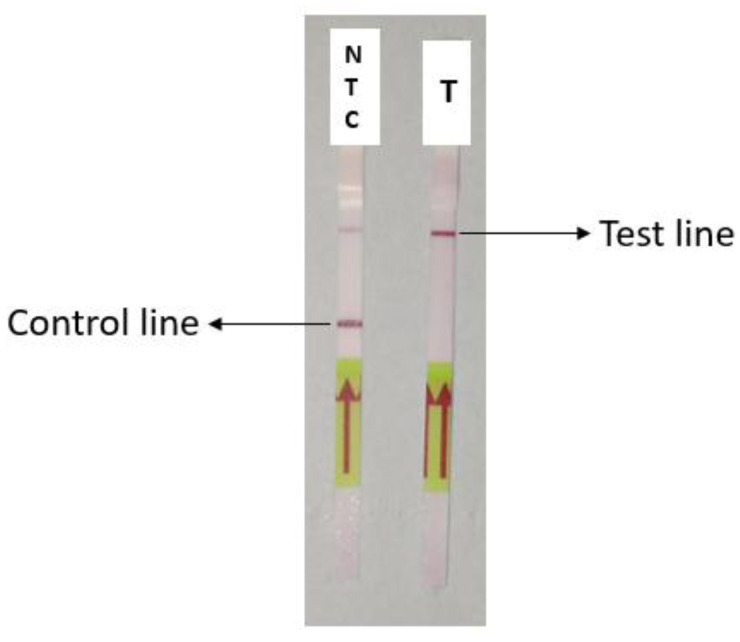
Lateral flow visualization of HCR/CRISPR-Cas12a. NTC: no template control, T: test sample with 5 µM of the target.

The HCR products with 5 µM of the probes and with (T) or without (NTC) the target were used to test the detection of SARS-CoV-2 using CRISPR-Cas12a. The sample containing the target demonstrated a strong band intensity at the test line. In contrast, the NTC displayed a band intensity at the control line with a faint band at the test line (Figure 3). 

### 3.4. Clinical Evaluation of HCR Reaction

**Figure 4:**
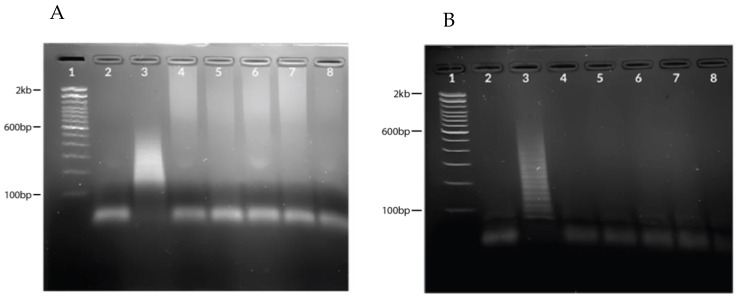
Gel image showing evaluation of clinical samples. (**A**) Hybridization chain reaction results using SARS-CoV-2 positive samples. Lane 1: 100 bp ladder, Lane 2: NTC, Lane 3: target, Lanes 4–8: positive samples (CT = 23.77, 27.96, 29.29, 31.07, 32.64, respectively). (**B**) Hybridization chain reaction results using SARS-CoV-2 negative samples. Lane 1: 100 bp ladder, Lane 2: NTC, Lane 3: target (positive control), Lanes 4–8: negative samples (CT = not detected for all samples).

HCR was performed on five positive and five negative samples, as confirmed by RT-qPCR. Using 6 μL of cDNA against 5 μM of H1 and H2 probes, each of the five positive samples showed a positive hybridization (Figure 4A), while the negative samples did not exhibit hybridization (Figure 4B). 

**Figure 5:**
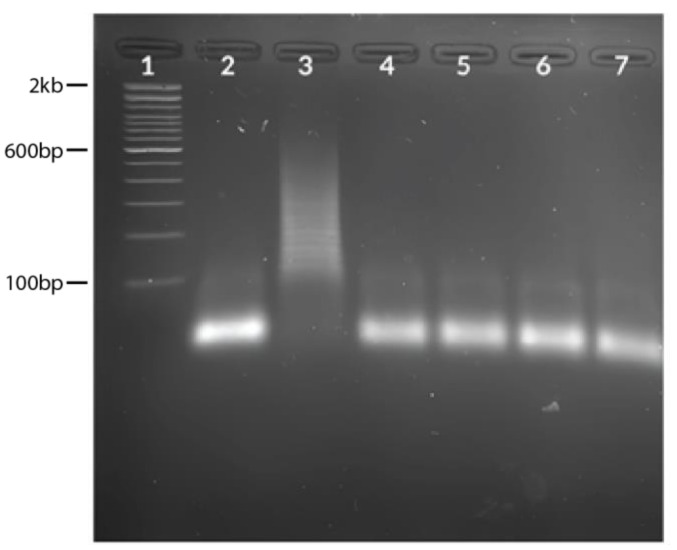
Specificity assay using seasonal flu virus samples. Lane 1: 100 bp ladder, Lane 2: NTC, Lane 3: target (positive control), Lane 4: H1N1pdm09, Lane 5: H3N2, Lane 6: B/Yamagata, Lane 7: B/Victoria.

Seasonal flu viruses (H1N1pdm09, H3N2, B/Yamata, and B/Victoria) were used for the specificity assay to check for possible cross-reactivity. The results showed no HCR amplification in H1N1pdm09, H3N2, B/Yamata, and B/Victoria except for the SARS-CoV-2 N gene target (Figure 5). 

## 4. Discussion

Reported prompt availability of the SARS-CoV-2 genetic sequences was important for the development of different confirmatory genetic-based COVID-19 diagnostic procedures, notably the real-time polymerase chain reaction (RT-PCR), which has been recognized as the benchmark standard molecular approach by the World Health Organization. However, the real-time polymerase chain reaction-based test is typically limited to laboratories and requires skilled personnel and specialized equipment. The limited facilities as well as the costs involved in performing the tests tend to burden centers for diagnosis in low- and middle-income countries, which affects planned batch testing processes and the preparation of laboratory reports and delays the transportation of results from central laboratories [8,9]. As a result, this study aimed to determine the viability of a diagnostic method that would be more appropriate in environments with limited resources. 

The HCR/CRISPR-Cas12a technology combined the polymerization efficiency of the hybridization chain reaction and the high specificity of CRISPR/Cas12a. The nucleic acid package (NUPACK), a web-based design algorithm, was used to design self-assembly reactions of HCR. The software designed and analyzed nucleic acids’ secondary structure in simulations involving different interacting strands [37]. Contrary to existing studies that use hybridization chain reaction and CRISPR on SARS-CoV-2 nucleic acids, the present study examined the application of the combined technology on SARS-CoV-2 samples.

The HCR probes were made to fold into the shape of a hairpin with overhangs to start the cascade of hybridization between the DNA hairpin probes and the single-stranded target at the beginning of the experiment. The probes were stable enough to maintain their hairpin shape in the absence of the target, but when the target was present, the hairpin probes facilitated the initiation of the hybridization chain reaction cascade. Incubation was carried out at 25 °C to help reduce interference with environmental factors. Other articles and reviews [34,38,39,40] have also highlighted the use of HCR for diagnostics, but few have reported its application in the clinical detection of SARS-CoV-2, which makes comparing the findings of this study difficult. However, similar results have been observed in a study that used algorithm-derived probes for the detection of the SARS-CoV-2 N gene using HCR [29].

In the study, the synthetic target sequences were subjected to CRISPR-Cas12a detection to verify the HCR. The lateral flow (LF) strip used for the HCR-Cas12a detection has biotin ligands and anti-FITC/FAM antibodies inserted into the control line (C-line) and test line (T-line). The 3′ and 5′ ends of the lateral flow reporter were labeled with biotin and FAM, respectively. The dual-labeled reporter, in which the FITC/FAM label is bound by mobile anti-FITC antibodies conjugated to gold nanoparticles (GNPs) and the biotin is bound by streptavidin, will remain intact if the LbCas12a-gRNA complex is unable to recognize the target DNA [35]. In such an incidence, a C-line with strong intensity and no T-line is seen. However, collateral cleavage occurs when the activated Cas proteins cut the dual-labeled reporter if the Cas complex recognizes the target DNA. A strong T-line intensity and/or a weak C-line intensity results from the separation of biotin and FITC/FAM labels caused by the cleavage. The appearance of the test line indicates that the results are positive [41], which was the case in our results.

When the target was not present, it was found during the experiment that the no-template control (NTC) had a weak T-line present. The presence of a faint T-line could be explained by the dose hook effect, where the amount of reporter added impacts the C- and T-line, which might cause misinterpretation of results, leading to false positive results [41]. However, the difference in band intensity indicated a distinction between the positive sample and the NTC. To ensure that this does not lead to false positives, we have found it convenient to always include a positive and negative sample in each test to ensure that the reader is well informed of the expected behavior of the test.

Evaluation of the assay using clinical samples showed successful hybridization in all the RT-PCR-confirmed positive samples, while no hybridization was detected in the RT-PCR-confirmed negative samples. Evaluation using more samples was limited by the scarcity of positive samples as the number of cases has significantly reduced. The best method for clinically identifying SARS-CoV-2 infection has been established using PCR [29]. However, HCR provides a resource-friendly alternative, although information about its application in identifying SARS-CoV-2 in clinical samples is limited. According to the observations, HCR may be as sensitive as PCR in detecting SARS-CoV-2 viral infection [34]. To ascertain the sensitivity and limit of detection of the HCR assay on SARS-CoV-2, clinical samples are necessary because the sensitivity of our HCR assay was established using a synthetic target. Furthermore, the combination of HCR/CRISPR-Cas12a and lateral flow strips could increase the sensitivity of the assay. 

A good reaction assay should be able to distinguish between viruses. Reports on the specificity of the N gene on SARS-CoV-2 from the Centers for Disease Control and Prevention (CDC) and other studies have shown the N gene’s high specificity rate to SARS-CoV-2 and lack of cross-reactivity with other coronaviruses and respiratory viruses [21,35,36]. Due to the unavailability of other coronaviruses, seasonal flu viruses were used instead. Seasonal flu viruses (H1N1pdm09, H3N2, B/Yamata, and B/Victoria) were used to test the specificity of the HCR reaction assay, and no cross-reactivity was found because no hybridization chain reaction took place. These viruses were used due to their common traits with coronavirus in infecting humans and they are associated with similar symptoms [42]. However, it is recommended that a specificity assay be carried out with other coronaviruses to help make a definitive conclusion ruling out any cross-reactivity with other related viruses.

The performance of the HCR/CRISPR-Cas12a detection method does not require sophisticated equipment such as thermocyclers, and, with minimal training, can be carried out by personnel. HCR reaction can also be visualized with other sensing technologies such as colorimetric, biosensors, etc., and is not limited to gel electrophoresis. The process of HCR may hence be economically friendly as the reagents needed are inexpensive compared to RT-PCR, which may make it ideal for resource-limited settings. However, further research is needed to enhance the development of HCR for SARS-CoV-2 rapid diagnosis. 

Furthermore, it has been observed that HCR/CRISPR-Cas12a-based diagnostic technologies can be more rapid and more robust, especially with the growing number of variants of concern (VOC) [43]. Apart from the testing being isothermal, the combined technology of HCR/CRISPR can be digitized through the fluorescent emission capability of CRISPR-Cas12a [16]. This fluorescence can be read out through mobile applications, which can make use of the technology at point-of-care facilities more fitting in resource-limited settings [44]. However, another limitation of this study is that it did not investigate the performance of the technology on different variants of concern. The design of the HCR H1 and H2 probes focused on the SARS-CoV-2 N gene, which is proposed to be more conserved and has been the target of many RT-PCR tests.

Scalable and user-friendly diagnostic methods must be employed in low- and middle-income countries (LMICs) with limited resources in order to respond to viral illnesses such as SARS-CoV-2 effectively. The development of sensitive and focused diagnostic tools, such as HCR/CRISPR-Cas12a, can improve disease tracking and surveillance efforts in off-the-grid locations while lessening the load on centralized testing centers. The ability of LMICs to respond to viral epidemics may be considerably improved by these developments in diagnostic technology, ultimately leading to better outcomes for global health.

## 5. Conclusions

From the results, we proved the possibility of a hybridization chain reaction (HCR) and clustered regularly interspaced short palindromic repeats (CRISPR)-Cas12a assay for detecting SARS-CoV-2 infection. It was observed from the study that HCR/CRISPR-Cas12a might match the efficiency of RT-PCR as a diagnostic technique for detecting SARS-CoV-2. The study recommends the use of a large sample size as well as conducting chi-square detection with the gold standard for detecting clinical samples and validation of the assay. The findings call for greater research into HCR/CRISPR-Cas12a, which may be a more suitable diagnostic technology for detecting SARS-CoV-2 in under-resourced populations in low- and middle-income nations, as there is still a need for screening and testing. The technology can also be researched for use against similar viral infections. 

## Figures and Tables

**Table 1:** HCR target, H1 and H2 hairpin probes, guide RNA for SARS-CoV-2 N gene, and reporter sequences.

Name	Sequence 5′-3′
Target	**GAACGCTGAAGCGCTGGGGGCAAA**
H1 Probe	**TTTGCCCCCAGCGCTTCAGCGTTC**AATGCGGAACGCTGAAGCGCTGGG
H2 Probe	**GAACGCTGAAGCGCTGGGGGCAAA**CCCAGCGCTTCAGCGTTCCGCATT
Grna	UAAUUUCUACUAAGUGUAGAUCCCCCAGCGCUUCAGCGUUC
Reporter	/56-FAM/TTATTATT/3Bio/

The underlined sequence shows the PAM sequence, bolded sequences indicate probe sequences complementary to the target, and the grayed sequences show target sequences in gRNA.

## Data Availability

The information backing up the study’s conclusions are available within the article.
